# Real-world evidence on siponimod treatment in patients with secondary progressive multiple sclerosis

**DOI:** 10.1186/s42466-022-00219-3

**Published:** 2022-11-07

**Authors:** Liesa Regner-Nelke, Marc Pawlitzki, Alice Willison, Leoni Rolfes, Sinem-Hilal Oezalp, Christopher Nelke, Tristan Kölsche, Melanie Korsen, Matthias Grothe, Sergiu Groppa, Felix Luessi, Sinah Engel, Gereon Nelles, Eckhard Bonmann, Holger Roick, Anke Friedrich, Philipp Knorn, Harald Landefeld, Zoltan Biro, Michael Ernst, Antonios Bayas, Martina Menacher, Katja Akgün, Christoph Kleinschnitz, Tobias Ruck, Tjalf Ziemssen, Refik Pul, Sven G. Meuth

**Affiliations:** 1 Department of Neurology, Medical Faculty, University Hospital Düsseldorf, Düsseldorf, Germany; 2 Department of Neurology, University Medicine Essen, Essen, Germany; 3 Center for Translational Neuro- and Behavioral Sciences, University Hospital Essen, Essen, Germany; 4 Department of Neurology, University Hospital Greifswald, Greifswald, Germany; 5 Department of Neurology, University Medical Center of the Johannes Gutenberg-University Mainz, Mainz, Germany; 6 NeuroMed Campus Hohenlind, Cologne, Germany; 7 Department of Neurology, Klinikum Köln, Cologne, Germany; 8 E/M/S/A Center for Neurology / Psychiatry / Neuroradiology, Singen, Germany; 9 Center for Outpatient Neurology, Essen, Germany; 10 Clinic for Neurology Selzer, Baiersbronn, Germany; 11 Center for Neurology, Psychiatry and Psychotherapy, Sinsheim, Germany; 12 Department of Neurology, Faculty of Medicine, University of Augsburg, Augsburg, Germany; 13 Center of Clinical Neurosciences, University Hospital Carl Gustav Carus, Dresden, Germany; 14 Department of Neurology, Heinrich-Heine University Duesseldorf, Moorenstraße 5, 40225 Duesseldorf, Germany

**Keywords:** Siponimod, Secondary progressive multiple sclerosis, Multiple sclerosis therapie, Real-wolrd data, Sphingosine 1-phosphate, Disease-modifying therapy

## Abstract

**Background:**

Therapeutic options targeting inflammation in multiple sclerosis (MS) have evolved rapidly for relapsing–remitting MS, whereas few therapies are available for progressive forms of MS, in particular secondary progressive MS (SPMS). The approval of siponimod for SPMS has allowed for optimism in the otherwise discouraging therapeutic landscape.

**Methods:**

We conducted a retrospective, multicenter, non-interventional study analyzing the efficacy and safety of siponimod under real-world conditions in 227 SPMS patients. According to the retrospective study framework, data was acquired at prespecified time points. Clinical readouts were assessed every three months. Disease progression was determined as increase in expanded disability status scale (EDSS), radiological progression, or the occurrence of new relapses under treatment. For safety analyses, adverse events (AE) and reasons for discontinuation were documented. The collected data points were analyzed at baseline and after 6, 12 and 18 months. However, data were predominately collected at the 6- and 12-month time points as many patients were lost to follow-up. In a group consisting of 41 patients, a more detailed investigation regarding disease progression was conducted, including data from measurement of cognitive and motoric functions.

**Results:**

Under siponimod therapy, 64.8% of patients experienced sustained clinical disease stability at 12 months. Out of the stable patients 21.4% of patients improved. Of the remaining patients, 31.5% experienced EDSS progression, 3.7% worsened without meeting the threshold for progression. Relapses occurred in 7.4%. Radiological disease activity was detected in 24.1% of patients after six months of treatment and in 29.6% of patients at 12 months follow-up. The in-depth cohort consisting of 41 patients demonstrated no substantial changes in cognitive abilities measured by Paced Auditory Serial Addition Test and Symbol Digit Modalities Test or motoric functions measured with Timed 25-Foot Walk, 100-m timed test, and 9-Hole Peg Test throughout the 12-month study period. Radiological assessment showed a stable volume of white and grey matter, as well as a stable lesion count at 12 months follow-up. AE were observed in nearly half of the included patients, with lymphopenia being the most common. Due to disease progression or AE, 31.2% of patients discontinued therapy.

**Conclusion:**

Treatment with siponimod had an overall stabilizing effect regarding clinical and radiological outcome measures. However, there is a need for more intensive treatment management and monitoring to identify disease progression and AE.

**Supplementary Information:**

The online version contains supplementary material available at 10.1186/s42466-022-00219-3.

## Introduction

Multiple sclerosis (MS) is a chronic inflammatory demyelinating disease of the central nervous system (CNS) comprising distinct subtypes. The relapsing–remitting course (RRMS) is characterized by acute flares of inflammation that often trigger neurological symptoms, with a variable degree of subsequent recovery but often minimal disease progression in the early stages of disease. Progressive forms of MS (PMS), including secondary progressive MS (SPMS), are associated with continuous clinical decline secondary to chronic CNS inflammation and diffuse neurodegeneration [16]. Acute inflammation may still occur in the progressive forms, which would, in the case of SPMS, be termed “active” SPMS according to the Lublin criteria [19]. Immune-modulating therapies that target acute inflammation have proven remarkably effective in RRMS, whereas PMS has remained notoriously difficult to treat—particularly those without superimposed inflammation [8, 11, 20]. In previous clinical trials using broad immunosuppressants, the already limited clinical benefits were outweighed by adverse events (AE) [4, 21, 22]. Although AE have generally continued to be a concern, the randomized, double-blind, placebo-controlled phase 3 EXPAND clinical trial powered the advent and approval of siponimod for SPMS [12]. Siponimod is a new-generation sphingosine-1-phosphate receptor (S1PR) modulator, which holds promise to exert anti-inflammatory as well as neuroprotective effects [13]. In the EXPAND study, siponimod met its primary endpoint of reduced risk of 3-month confirmed disability progression (CDP). Notably, in this trial, the frequency of AE was just slightly higher in siponimod-treated patients (89%) than in the placebo group (82%), which was also observed for serious AE (SAE) (15% vs. 18%) [12]. Given that patients in the EXPAND study differ from real-world populations regarding both individual and disease-specific characteristics, this may lead to limited generalizability of results in standard clinical practice [25]. Thus, investigation of treatment efficacy and occurrence of AE in a real-world cohort of SPMS patients is required to provide robust information, so clinicians and patients know what to expect when considering this treatment option. Here, we present the results of a retrospective, multicenter study analyzing the efficacy and safety of siponimod in SPMS patients under real-world conditions.

## Methods

### Study design

**Fig. 1:**
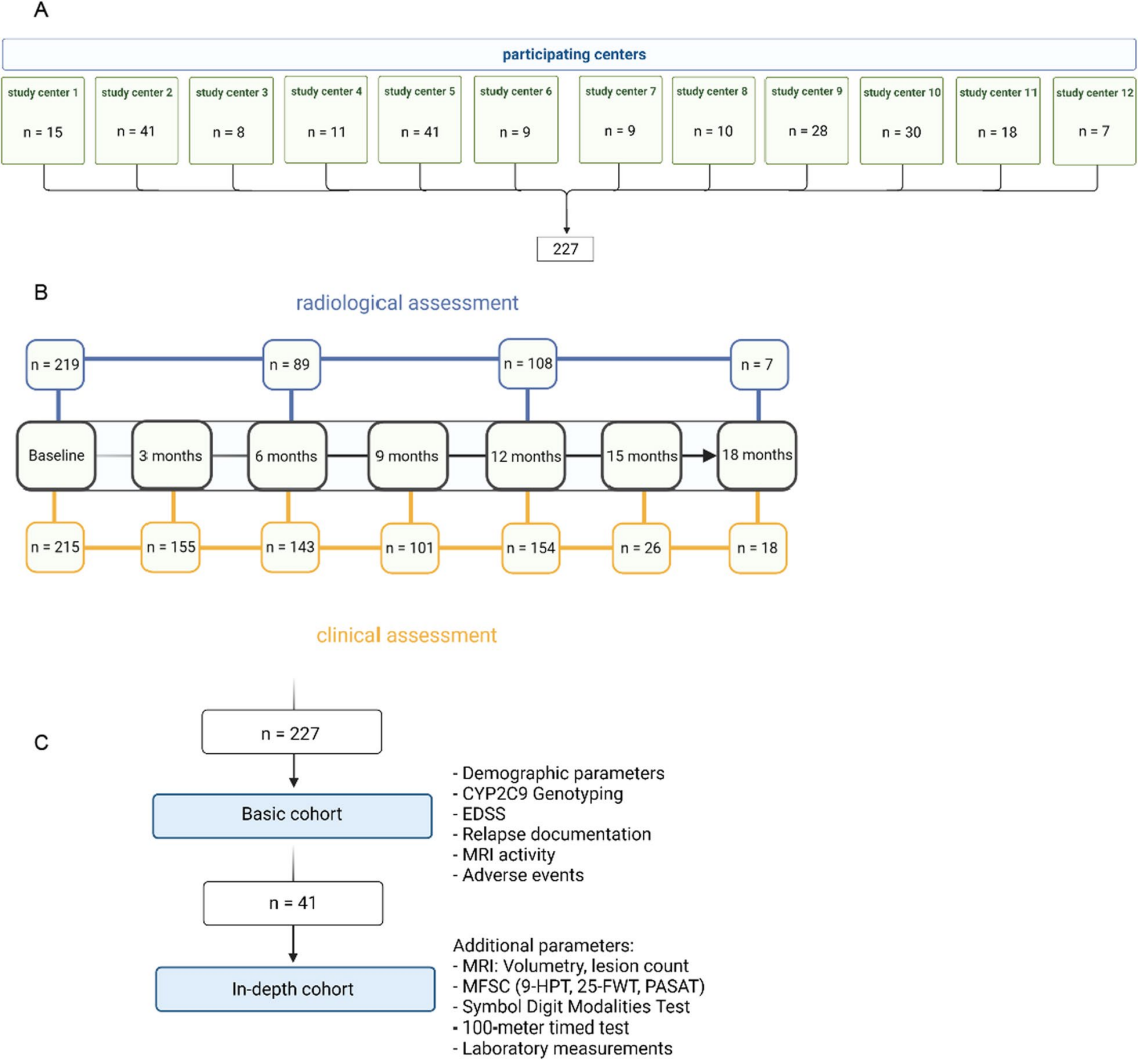
Study design. Part **A** demonstrates the patients contributed by participating centres. Part **B** illustrates the timeline of clinical and radiological assessment. Part **C** depicts the two cohorts included in the study, with the additional parameters only being used in the in-depth cohort

We conducted a retrospective, observational study that included SPMS patients receiving siponimod in selected sites in Germany. Study centers were selected according to local requirements for non-interventional studies. Physicians practicing in neurological in- and outpatient clinics in Germany were eligible to take part in this study (Fig. 1A). Inclusion criteria were age equal or greater than 18 years and diagnosis of SPMS [18]. Collected data included clinical, epidemiological, and disease-specific characteristics, i.e. disease duration (timespan between diagnosis of RRMS and baseline examination), number and type of previous therapy, and relapse rate. Data was collected during clinical assessments and follow-up visits as a part of routine clinical practice. The data points were assigned to fit the study design with readouts every three months (Fig. 1B). In the case of discontinuation of therapy, a further readout 3 months after discontinuation was done.

Brain magnetic resonance imaging (MRI) analyses had to be limited to follow-up examinations at months 6 and 12 since MRI data were scarcely available for earlier or later time points.

To analyze whether age at baseline influences disease outcome, patients were stratified by age over or under 50 years. To evaluate the impact of disease duration, defined as the time since initial diagnosis, patients were classified into groups of shorter or longer disease duration using the mean disease duration of the cohort [17.8 years, standard deviation (SD) 9.4] as the separator. To analyze the impact of baseline disability, baseline Expanded Disability Status Scale (EDSS) ≤ 4 or > 4 served for grouping. Disease progression was present if at least one of the following conditions was true: (a) the increase of 0.5 or 1.0 points on the EDSS if baseline EDSS was > 5.5 or ≤ 5.5, respectively; (b) radiological progression as defined as one new cerebral T2 lesion; (c) the occurrence of relapses under treatment. Disease progression analysis were conducted only for patients with available clinical examination and MRI data 12 months after baseline. For safety analyses, AE and reasons for discontinuation were documented.

In a smaller group consisting of 41 patients, a more detailed analysis regarding disease progression was conducted (Fig. 1C). In this in-depth cohort, data from the Timed 25-Foot Walk (T25FW) test [9], 100-m timed test [2], 9-Hole Peg Test (9HPT) [6], Paced Auditory Serial Addition Test (PASAT) [7], and Symbol Digit Modalities Test (SDMT) [23] were collected. Regarding laboratory data, the lymphocyte count and subgroup (CD4^+^ and CD8^+^ T cells, natural killer (NK) and B cells) were assessed via standard hematology laboratory measures. Lymphocyte subsets were assessed in a central laboratory using flow cytometry.

### Radiological assessment

MRI imaging from patients was performed using non-standardized protocols from different MRI units and magnetic field strengths (1.5 or 3.0 Tesla). Imaging ideally occurred at baseline and after 6, 12 and 18 months. All MRI protocols included T1- and T2-weighted spin-echo sequences. Abnormalities including T1-hypointensities, T2-hyperintensities, T1-lesions were identified by an experienced MS specialist.

Additional MRI measurements were carried out in the in-depth cohort at baseline and after 6 and 12 months. Brain volume measurements were conducted using volBrain (http://volbrain.upv.es), a publicly available online MRI brain volumetry system. Based on multi-atlas label fusion technology, grey matter volume (GMV) including cortical and subcortical and cerebellar structures and white matter volume (WMV) were calculated. Lesions volume was analyzed in absolute (cm^3^) and normalized (%) volume. Lesion number was also examined using volBrain.

### Statistics

All data were analyzed using GraphPad Prism version 9.3.1. Sunburst diagrams were created using Windows Excel Version 2013. Qualitative variables were described using absolute or relative frequencies. Quantitative variables were presented using the mean with the SD or median with the interquartile range [IQR]. The collection of data regarding disease progression was classified into three-month episodes in line with standard clinical practice for follow-up visits. Missing data were not included in the analyses. T-test and the Mann–Whitney U-test for non-parametric variables were used. For the correlation analysis, the Pearson correlation coefficient was determined. *P* values ≤ 0.05 were deemed to be statistically significant.

### Ethical approval

Before trial initiation, an ethics committee was consulted and approved the retrospective analysis of clinical data in the Department of Neurology of the University of Duesseldorf, including the data that was analyzed in this study (No. 5794R). The study was conducted in accordance with the Declaration of Helsinki.

## Results

### Study population

**Fig. 2:**
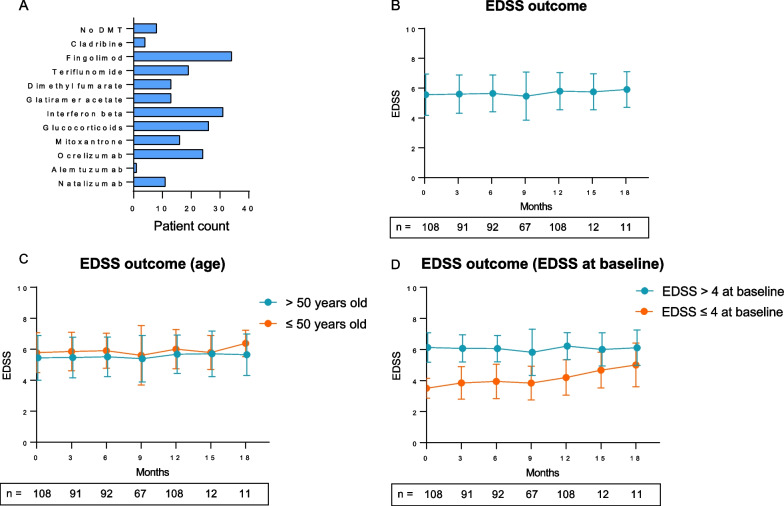
Patient characteristics and EDSS outcome. Part **A** demonstrates the number of previous DMTs per patient prior to siponimod treatment. Part **B** shows the number of patients at each point in follow-up and the corresponding mean EDSS at that time. Part **C** demonstrates the cohort divided into age above and below 50 and associated EDSS over time, with the number of patients at each time point of follow up given. Part **D** displays the EDSS over time with patient number at each follow-up point included, with the cohort divided into EDSS ≤ 4 or > 4. EDSS, Expanded Disability Status Scale

A total of 227 patients with SPMS treated with siponimod were included in this study. The mean age at baseline was 53.4 (SD ± 8.5) years, with a slight female predominance (56.0%). The mean disease duration since diagnosis of MS to baseline was 15.8 (SD ± 9.4) years. Median EDSS at baseline was 6.0. Regarding previous disease modifying therapies (DMTs), with fingolimod being the most common previous medication, 8 patients were treatment-naïve (Fig. 2A). The mean number of previous DMTs was 2.5 (SD ± 1.9). Genotyping before therapy initiation in accordance with clinical guidelines demonstrated that most patients (77.9%) carried the cytochrome P2C9*1*2- (CYP2C9*1*2-) and-*1*1-genotype and therefore received a maintenance dose of 2 mg per day. 22.1% of patients with the CYP2C9*2*3- or *1*3- genotype received the recommended dose of 1 mg per day.

### Efficacy outcomes

#### Disability progression

Disability progression analysis was conducted only for patients with a complete baseline and 12 moth follow up (n = 108). In this cohort of 108 patients the mean age at baseline was 53.4 (SD ± 9.1), with 53.7% woman. The mean disease duration at baseline since diagnosis of MS was 17.8 (SD ± 9.3) years. Median EDSS at baseline was 6.0. The mean number of previous DMTs was 2.6 (SD ± 1.7), with fingolimod being the most common one. 80.4% of patients carried CYP2C9*1*2- and-*1*1-genotype and 19.6% of patients carried the CYP2C9*2*3- or *1*3- genotype.The average number of relapses per year prior to siponimod initiation ranged from 0 to 3 with a mean of 0.4 (SD ± 0.7). 8 patients (7.4%) experienced relapses under siponimod treatment over the observational time period. There was no significant association between sex (*p* = 0.45) or age (*p* = 0.59) of the participants and occurrence of relapses. At 12 months, 70 patients (64.8%) experienced sustained disease stability. Out of the stable patients 15 (21.4%) improved. 34 patients (31.4%) experienced EDSS progression and 4 patients (3.7%) worsening in the EDSS without meeting the threshold for progression. However, we did not find any relationship to prior relapse activity before siponimod initiation. Prior to siponimod initiation 10 patients (9.3%) with a baseline MRI showed new contrast enhancing lesions, 29 patients (26.9%) showed new T2 lesions. Of the patients that remained stable under siponimod 21 patients (30.0%) showed a stable MRI prior to siponimod initiation. Overall, at 12 months (*p* = 0.2, n = 108) no significant changes in the EDSS score were observed (Fig. 2B). When classifying patients into a group of shorter and longer disease duration, we observed a marked, but not statistically significant lower EDSS in patients with shorter disease duration at 12 months of follow-up (*p* = 0.09). No difference was observed when comparing patients younger or older than 50 years (*p* = 0.14) (Fig. 2C). When comparing EDSS at baseline, the group with EDSS > 4 at baseline remained stable, but the EDSS ≤ 4 group demonstrated a significant increase in EDSS from baseline to 12 months (*p* = 0.01) (Fig. 2D).

**Fig. 3:**
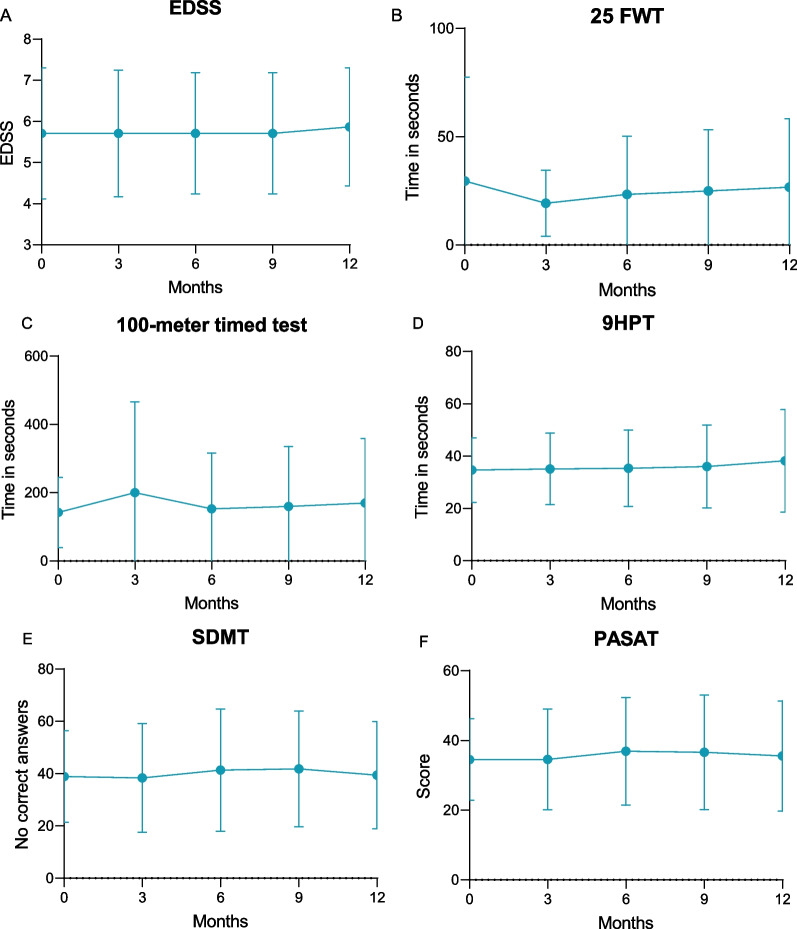
Measurements of therapeutic efficacy over time. EDSS (part **A**), 25 FWT (part **B**), 100-m time test (part **C**), 9 HPT (part **D**), SDMT (part **E**), PASAT (part **F**) are stable over time. EDSS, Expanded Disability Status Scale; 25 FWT, 25-timed food walking time test; 9HPT, 9-hole peg test; SDMT, Symbol Digit Modalities Test; PASAT, Paced Auditory Serial Addition Test

The in-depth cohort consisting of 41 patients demonstrated no significant change in EDSS throughout the first 12 months after treatment initiation (Fig. 3A). Similarly, no change was observed in the assessment of motoric functions with T25FW, 100-m time test or 9HPT (Fig. 3B–D). Testing of cognitive abilities through PASAT and SDMT were also stable throughout the 12 months of follow-up (Fig. 3E, F). With regard to differences associated with baseline EDSS, we did not observe relevant changes for T25FW, 100-m time test, 9HPT PASAT and SDMT in the in-depth cohort (Additional file 1: Fig. S1).

### MRI-monitored disease progression

Radiological disease activity was detected in 26 patients (24.1%) after 6 months of treatment and in 32 patients (29.6%) at 12 months follow-up. The in-depth cohort showed a stable volume of white (*p* = 0.35) and grey matter (*p* = 0.47) throughout the first 12 months of treatment. Lesion count (*p* = 0.74) and volumetry (*p* = 0.29) also did not change significantly within the first 12 months of treatment. Radiological disease activity did not correlate with previous immunotherapies.

### Safety outcomes

**Fig. 4:**
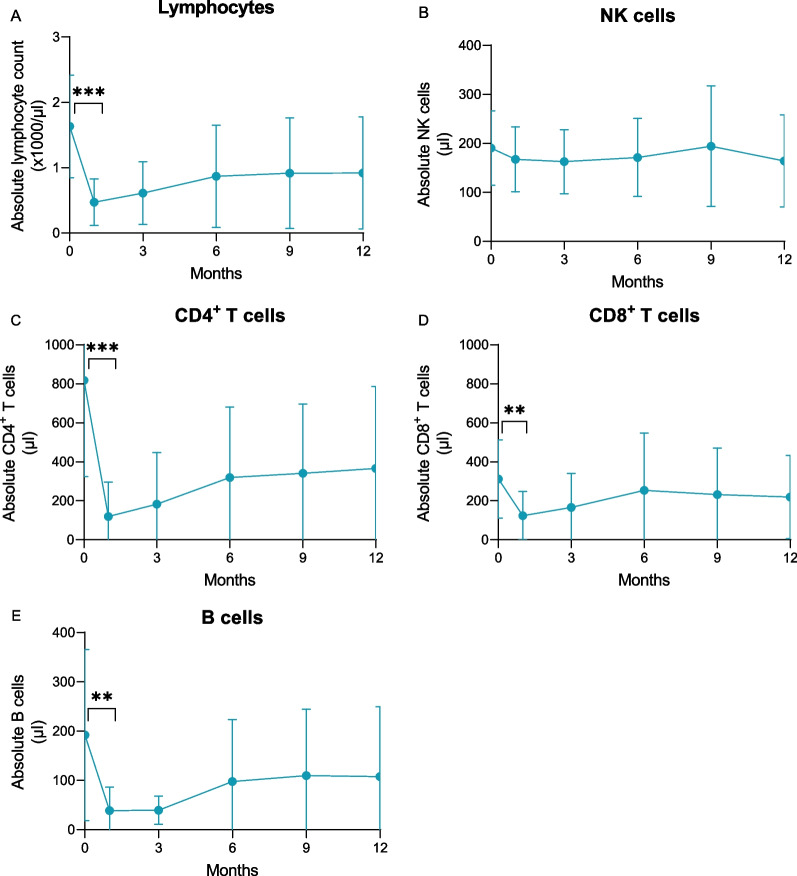
Measurements of immune cells over time. A decline in absolute lymphocyte count as well as all lymphocyte subgroups was observed within the first month of treatment (total lymphocyte count, part **A**; NK cell count, part **B**; CD4^+^ T cell count, part **C**; CD8 + T cell count, part **D**; B cell count, part **E**), with significant differences in absolute lymphocyte count (*p* ≤ 0.001), CD4^+^ T (*p* ≤ 0.001), CD8^+^ T (*p* ≤ 0.01) and B cells (*p* ≤ 0.01). In the following months of treatment, an increase in lymphocyte count occurred but remained below baseline (part **A**). CD4^+^ T cells remained significantly below baseline throughout the first 9 months (part **C**). B cells stayed significantly below baseline throughout the first 3 months (part **E**). NK cells, natural killer cells

A decline in absolute lymphocyte count as well as all lymphocyte subgroups was observed within the first month of treatment, with significant differences in absolute lymphocyte count (*p* ≤ 0.001), CD4^+^ T (*p* ≤ 0.001), CD8^+^ T (*p* ≤ 0.01) and B cells (*p* ≤ 0.01) except NK cells (*p* = 0.40). In the following months of treatment, an increase in lymphocyte count occurred but remained below baseline (Fig. 4A–E). CD4^+^ T cells remained significantly below baseline throughout the first 9 months. Lymphocytes in general as well as B cells remained significantly below baseline throughout the first 3 months. We did not observe any relation between previous immunotherapies and clinical outcomes (data not shown).

Of the 227 patients included in this study, 31.2% discontinued therapy over the entire observational period of 18 month. There was no significant association between sex (*p* = 0.69) or age (*p* = 0.55) and the decision to discontinue therapy. The main reason for discontinuation was the experience of AE (62.6% of patients who discontinued therapy), with patient wish (2 patients), disease progression (7 patients), inefficacy (4 patients), concern regarding side effects (4 patients), refusal of MRI monitoring (1 patient), death due to unknown reason (1 patient), or unknown reason for discontinuation (2 patients) associated with discontinuation in other cases.

Patients with an available follow up 3 months after discontinuation showed relapse activity in 8 patients (11.9%) and new T2 lesions in 19 patients (28.4%). When comparing the last documented EDSS under treatment with the EDSS 3 months after discontinuation of siponimod-therapy, there was seen no significant difference (*p* = 0.87, n = 47).

**Fig. 5:**
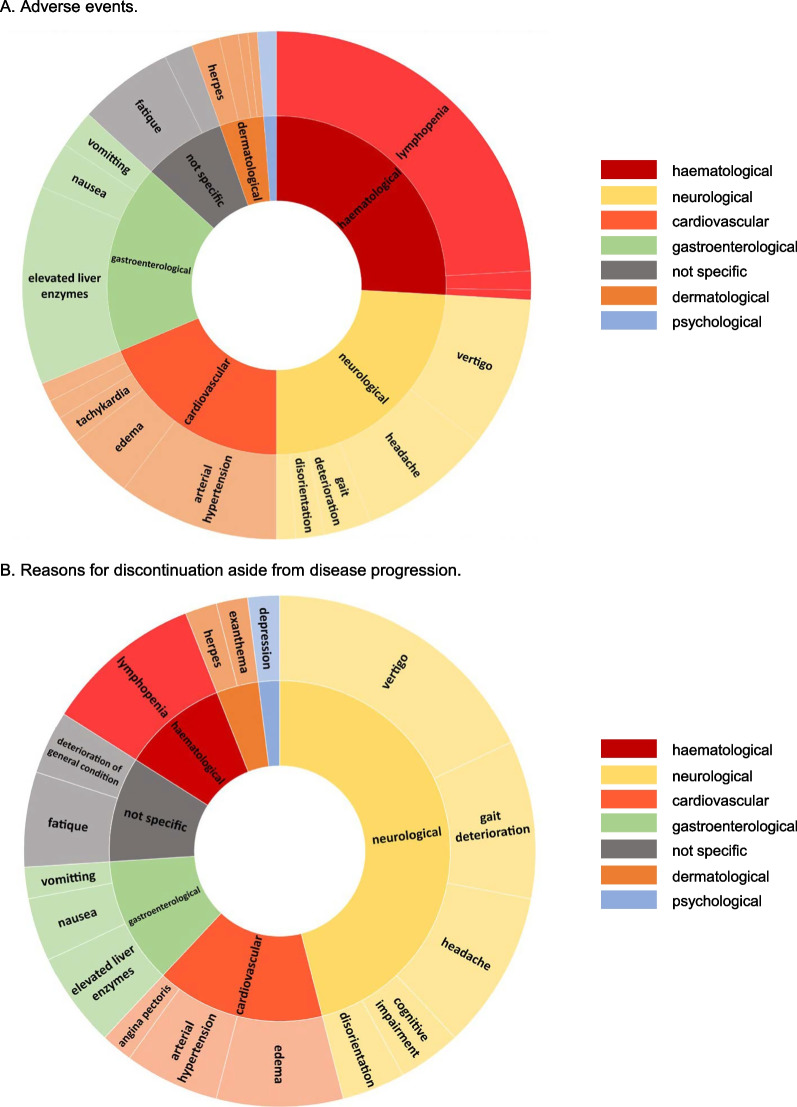
Adverse event (AE) profile and reasons for discontinuation. Part **A** AE occured in 46.0% of patients, with lymphopenia being the most common (38.1%). This was followed by elevated liver enzymes (20.0%) and arterial hypertension (16.2%). Part **B** The most common AE being a reason for discontinuation was vertigo (15.2%). When comparing AE in general and AE that led to discontinuation, a different distribution was observed. While hematological AE were the most common group in general AE, AE that manifested as neurological symptoms were the most common AE that led to discontinuation of therapy. AE, adverse events

AE occurred in 47.9% of patients, with lymphopenia being the most common (38.1%), followed by elevated liver enzymes (20.0%) and arterial hypertension (16.2%). Interestingly, 26.8% of all patients reporting AE continued therapy. There was no association between the occurrence of AE and sex or CYP2C9 genotype (data not shown). While hematological AE were the most common in general, AE manifesting as neurological symptoms were the most common AE that led to discontinuation of therapy (Fig. 5A, B). The most common AE being a reason for discontinuation of therapy was vertigo (15.2%). Of note, previous immunotherapy did not influence the occurrence of AEs.

## Discussion

In the present study, we analyzed the efficacy, the adverse event profile and discontinuation rate of siponimod as treatment for SPMS in a real-world cohort. During this short-term observation with only a few patients participating in follow ups after 12 months, treatment with siponimod had an overall stabilizing effect regarding clinical and radiological outcome measurements. However, both disease activity and the occurrence of AE led to the discontinuation of siponimod in a relevant proportion of patients.

In this study, with a maximum observation period of 18 months, we documented a higher rate of EDSS progression compared with the treatment arm of the EXPAND study. Interestingly, EDSS alterations were similar in patients aged 50 and older compared to younger patients, which is in line with a recent post-hoc analysis of the EXPAND cohort [10]. Surprisingly, when considering less severely affected patients with an EDSS of 4 or lower in our cohort, we observed a slight but significant EDSS worsening after 12 months of treatment compared to baseline. A caveat to this observation is that the study population decreased due to discontinuation or incompliance during the follow-up period. Interestingly, severely affected patients were stable under siponimod as shown in the EXPAND trial that included a high proportion of severely disabled patients, with 56% of patients having a baseline EDSS of 6 or more [12]. Another explanation might be that the EDSS is less sensitive and later indicative to progression in PMS patients with a high baseline score [15]. Consequently, disease progression and treatment efficacy may be captured inadequately in patients with higher EDSS at baseline. Moreover, when considering clinical outcomes in SPMS, EDSS appears to be more prone to individual variation than T25FW or 9HPT [14]. Therefore, time to 3-month confirmed worsening in the T25FW of at least 20% from baseline served as a secondary endpoint in the EXPAND trial [12]. Consequently, we included T25FW and 9HPT readouts in the in-depth cohort of our study and observed stable values throughout the observation period. Consistent with this, recent data from the open-label extension phase of the EXPAND trial suggest sustained clinical achievement over 5 treatment years as well as a significant risk reduction of disease progression for patients in the active treatment arm from the start compared to patients who had started with placebo treatment and switched to siponimod [5]. As in the EXPAND trial, we did not observe any deterioration in SDMT [3].

However, radiological disease activity measured by new T2 was observed in 29.6% of our cohort at month 12, which might suggest that subclinical inflammatory disease progression occurred in a proportion of patients despite treatment. Given the high proportion of highly effective prior DMTs in our cohort compared to the EXPAND trial [12], it might be the case that some of the SPMS patients still have persistent inflammatory disease activity that siponimod fails to sufficiently suppress. Thus, treatment switch from highly active DMT to siponimod should be closely monitored to detect inflammatory activity during the washout period. Volumetric analyses of 41 patients from the in depth cohort at 6 and 12 months follow-up did not show any meaningful reduction of brain tissue within our short observation period, which potentially underline the treatment efficacy of siponimod as well. In line with this, advanced radiological analyses of the EXPAND cohort indicate improvement of brain tissues integrity under siponimod therapy [1].

In terms of safety analyses, studies including PMS patients, especially those over 50 years of age, are complicated by AE [24]. In our study, AE were reported in almost 50% of patients. The frequencies of certain AE showed to be different than reported in the EXPAND trial, where infections and infestations seemed to be the major problem, followed by cardiovascular events like arterial hypertension and liver-related problems. In our cohort, lymphopenia was the most common AE, while lymphopenia only accounted for ~ 1% of AE in the EXPAND trial [12]. Moreover, the occurrence of AE was the main reason for treatment discontinuation. As discontinuation of therapy occurred more frequently in our cohort compared to the EXPAND trial, AE constitute a substantial challenge for treatment adherence and management in the real-world setting. It is worth noting that 11.9% patients experienced relapse activity and 28.4% of patients demonstrated new T2 lesions in the following three months after discontinuation. Together, these data underpin the risk for disease flare-ups in response to treatment discontinuation for patients receiving siponimod.

A limitation of our study is that a significant number of patients were lost to follow-up, which was most pronounced after 12 months. Therefore, regarding MRI, only a short observation period of 12 months was possible. Regarding EDSS scores, the loss to follow-up might lead to less reliable data at certain time points. Moreover, the assessment of MRI outcomes was done by MS specialists instead of neuroradiologists.

## Conclusion

In summary, we provide real-world data on the efficacy of siponimod in SPMS patients. In light of our real-world efficacy and safety data, clinicians should be aware that patients must be closely monitored, particularly those with an EDSS ≤ 4 and/or age < 50 years because of disease progression and, in general, because of a high frequency of early AE that might lead to treatment discontinuation. Moreover, discontinuation of treatment might be associated with a recurrence of disease activity, requiring close clinical and radiological monitoring. Future long-term real-world studies will provide more clarity as to exactly which patients will benefit from the siponimod treatment [17].

## Supplementary Information

**Additional file 1: Figure S1.** Measurements of therapeutic efficacy over time divided according to EDSS. EDSS (part A), 25 FWT (part B), 100-m time test (part C), 9 HPT (part D), SDMT (part E), PASAT (part F) were not significantly different when groups were separated into EDSS ≤ 4 or > 4. EDSS, Expanded Disability Status Scale; 25 FWT, 25-timed food walking time test; 9HPT, 9-hole peg test; SDMT, Symbol Digit Modalities Test; PASAT, Paced Auditory Serial Addition Test.

## Data Availability

The datasets used and analysed during the current study are available from the corresponding author on reasonable request.

## Abbreviations

AE: Adverse events
CDP: Confirmed disability progression
CNS: Central nervous system
Cm3: Square centimetres
CYP2C9: Cytochrome P2C9
DMT: Disease modifying therapy
EDSS: Expanded Disability Status Scale
Gd: Gadolinium
GMV: Grey matter volume
IQR: Interquartile range
Mg: Milligram
MS: Multiple sclerosis
MRI: Magnetic resonance imaging
NK: Natural killer cells
PASAT: Paced Auditory Serial Addition Test
PMS: Progressive multiple sclerosis
RRMS: Relapsing–remitting multiple sclerosis
SAE: Serious adverse events
SD: Standard deviation
SDMT: Symbol Digit Modalities Test
SPMS: Secondary progressive multiple sclerosis
T25FW: Timed 25-Foot Walk
WMV: White matter volume
9HPT: 9-Hole Peg Test
