# Supplementary figures and images for: Real-world evidence on siponimod treatment in patients with secondary progressive multiple sclerosis
Source: Neurol Res Pract. 2022 Nov 7;4:55. doi: 10.1186/s42466-022-00219-3 (PMC9639325; doi:10.1186/s42466-022-00219-3)

## Slide 1
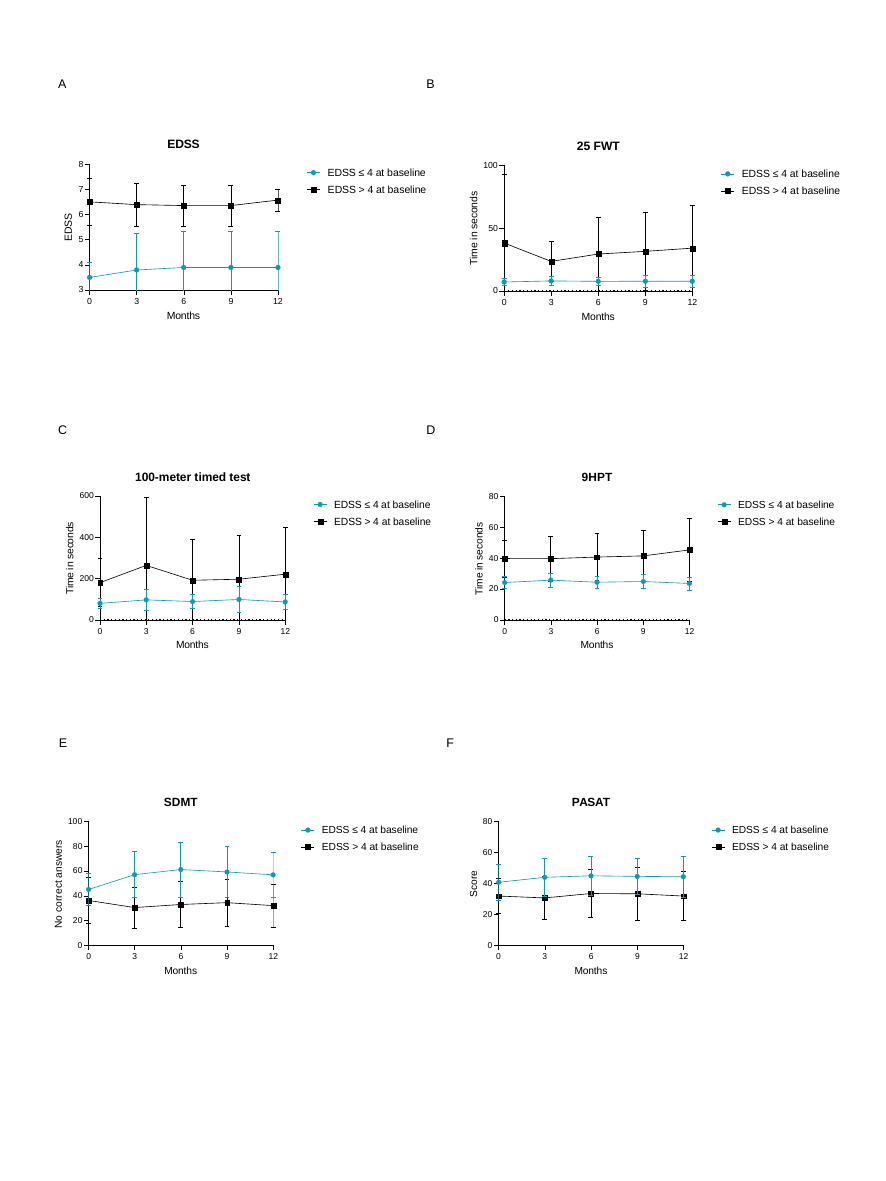

A
B
C
D
F
E

Supplement: Supplementary file 1 — Additional file 1: Figure S1. Measurements of therapeutic efficacy over time divided according to EDSS. EDSS (part A), 25 FWT (part B), 100-m time test (part C), 9 HPT (part D), SDMT (part E), PASAT (part F) were not significantly different when groups were separated into EDSS ≤ 4 or > 4. EDSS, Expanded Disability Status Scale; 25 FWT, 25-timed food walking time test; 9HPT, 9-hole peg test; SDMT, Symbol Digit Modalities Test; PASAT, Paced Auditory Serial Addition Test. [file 42466_2022_219_MOESM1_ESM.ppt]
